# Oral rehydration of malnourished children with diarrhoea and dehydration: A systematic review

**DOI:** 10.12688/wellcomeopenres.12357.3

**Published:** 2017-10-27

**Authors:** Kirsty A. Houston, Jack G. Gibb, Kathryn Maitland

**Affiliations:** 1 Department of Paediatrics, Faculty of Medicine, Imperial College, London, W2 1PG, UK; 2 KEMRI-Wellcome Trust Research Programme, Kilifi, 80108, Kenya

**Keywords:** malnutrition, gastroenteritis, dehydration, rehydration, systematic review, Africa, Asia, oral rehydration solution

## Abstract

**Background**: Diarrhoea complicates over half of admissions to hospital with severe acute malnutrition (SAM). World Health Organization (WHO) guidelines for the management of dehydration recommend the use of oral rehydration with ReSoMal (an oral rehydration solution (ORS) for SAM), which has lower sodium (45mmols/l) and higher potassium (40mmols/l) content than old WHO ORS. The composition of ReSoMal was designed specifically to address theoretical risks of sodium overload and potential under-treatment of severe hypokalaemia with rehydration using standard ORS. In African children, severe hyponatraemia at admission is a major risk factor for poor outcome in children with SAM complicated by diarrhoea. We therefore reviewed the evidence for oral rehydration therapy in children with SAM.

**Methods**: We conducted a systematic review of randomised controlled trials (RCTs) on 18 ^th^ July 2017 comparing different oral rehydration solutions in severely malnourished children with diarrhoea and dehydration, using standard search terms. The author assessed papers for inclusion. The primary endpoint was frequency of hyponatraemia during rehydration.

**Results**: Six RCTs were identified, all published in English and conducted in low resource settings in Asia. A range of ORS were evaluated in these studies, including old WHO ORS, standard hypo-osmolar WHO ORS and ReSoMal. Hyponatraemia was observed in two trials evaluating ReSoMal, three children developed severe hyponatraemia with one experiencing convulsions. Hypo-osmolar ORS was found to have benefits in time to rehydration, reduction of stool output and duration of diarrhoea. No trials reported over-hydration or fatalities.

**Conclusions**: Current WHO guidelines strongly recommend the use of ReSoMal based on low quality of evidence. Studies indicate a significant risk of hyponatraemia on ReSoMal in Asian children, none have been conducted in Africa, where SAM mortality remains high. Further research should be conducted in Africa to evaluate optimal ORS for children with SAM and to generate evidence based, practical guidelines

## Introduction

In Africa, diarrhoea has been reported to complicate 49% of admissions to hospital of children with severe acute malnutrition (SAM), and a further 16% develop diarrhoea within 48 hours of admission. The in-hospital case fatality in children with SAM admitted with diarrhoea is high, 19%, versus 9% in those without diarrhoea, (χ ^2^  =  17.6 p<0.001) and no prospect of improvement has been demonstrated over the last decade ^1– 4^.

Management of children with SAM complicated by diarrhoea focuses on exclusive oral or nasogastric (NG) rehydration, and limits intravenous rehydration to those complicated by advanced hypovolaemic shock or for those with severe dehydration who are unable to take or tolerate oral fluid ^5, 6^. The World Health Organization (WHO) guidelines are used widely in low resource settings as the standard of care, recommending that oral or NG rehydration fluids can be commenced for any child with SAM and diarrhoea (defined as three or more loose, watery stools) ( Table 1). The guidelines do not allow for an assessment of severity of dehydration in children with SAM, indicating that dehydration is often difficult to diagnose in malnourished children because the clinical signs usually relied on to diagnose dehydration are similar to those found in severe wasting without dehydration ^5^. However, the available evidence contradicts this contention. In a prospective study involving 920 unselected Kenyan children admitted to hospital with SAM, sepsis, signs of severe dehydration (secondary to diarrhoea) and hypovolaemic shock were common complications and were triage features associated with high early fatality (>20% mortality) ^7^. Another prospective observational study conducted at the same centre examined in more detail diarrhoea in malnutrition, and multivariate analysis identified bacteraemia (odds ratio 6.7 (95% confidence interval 2.5-17.8 p<0.001) and hyponatraemia (odds ratio 4.9 (95% CI 2.2-11.1 p<0.001) as key risk factors for mortality ^1^. Only a very small number of children with signs of advanced shock are recommended to receive intravenous (IV) fluids, 15ml/Kg of hypotonic fluid, followed by a blood transfusion if there is no improvement ( Table 1). Outcomes in this group remains very unsatisfactory (reviewed by Houston *et al.,* 2017) ^8^

**Table 1.** Current recommendations for treatment of severely malnourished children with severe dehydration (WHO 2013) ^6, 12^.

	No shock	Shock *
Initial	ReSoMal PO/NG – 5ml/kg every 30 minutes for first 2 hours	15ml/Kg 1/2SD+5% **OR** RL+5%, over 1 hour, repeated once if needed If no improvement: Transfusion 10ml/Kg over 3hours (start 4ml/Kg/hour maintenance while awaiting blood)
Subsequent	Then 5–10ml/kg/hr, alternating F75 and ReSoMal for 4–10 hours	Oral or nasogastric ReSoMal alternating with F75 10ml/Kg/hr, up to 10hrs, and then refeeding with F75

*Shock is defined as the presence of all three of the following signs: Prolonged capillary refill time (>3seconds), temperature gradient and weak and fast pulse. ReSoMal – rehydration solution for malnutrition, PO/NG – Oral or nasogastric route, RL+5% – Ringers lactate and 5% dextrose, 1/2SD+5% - ½ strength Darrow’s solution and 5% dextrose, F75 – primary feeding formula for children with SAM

Recommendations suggest avoiding IV fluids in children with SAM due to concerns about the ability of these children to handle significant volume loads and potential susceptibility to fluid overload and cardiac failure. However, available evidence suggests that the perturbations of myocardial function are related to complications of sepsis, shock and severe dehydration and not due to ‘heart failure’ ^9, 10^. A recent publication by Obonyo *et al.* 2017 demonstrated ‘fluid responsive’ myocardial indices following rehydration in children with SAM and hypovolaemic shock ^11^.

### Types of Oral Rehydration Solutions

The original or ‘old’ oral rehydration solution (ORS) recommended by WHO was designed largely to treat children with cholera and thus had a high sodium content (since cholera is a secretory diarrhoea with large losses of both sodium and water). At that time WHO guidance advised that children with SAM should be given a modified version of oral rehydration solution (ORS) called ReSoMal (rehydration solution for malnutrition), which has lower sodium, higher potassium and glucose and lower osmolarity than Old WHO ORS ( Table 2) ^5, 6^. This was due to concerns that ‘children with bilateral pitting oedema typically have high intracellular sodium and are therefore inclined to retain fluids’ and ‘are prone to fluid retention and susceptible to fluid changes’, thereby predisposing the child to fluid overload and heart failure ^6^. Whilst this was suggested by *Wharton et al.*, who reported an excess of heart failure in children receiving a high energy milk to which sodium was added ^13^, apparently to improve acceptability in all children (including those without diarrhoea) the sodium content was probably higher and the milk given over a longer period of time than they would have received if they were only being rehydrated. Thus, this cannot be extrapolated to inform management of rehydration in children with SAM. Furthermore, an observational study has reported that in SAM children receiving liberal ReSoMal leads to excess mortality due to heart failure due to excessive sodium intake as a reference source to support these recommendations. However, this does not qualify as sufficient evidence to inform the management of African children who have diarrhoea ^14^. Other than these observations we are have found no physiological data published to support this contentious opinion that children with severe malnutrition are prone to sodium overload, even for the sub group of greatest concern, kwashiorkor ^15, 16^.

**Table 2.** Comparison of formulations of oral rehydration solution (ORS).

	Old WHO ORS	Standard hypo-osmolar WHO ORS	ReSoMal
Osmolarity (mOsm/L)	311	245	300
Sodium Mmol/l	90	75	45
Potassium Mmol/l	20	20	40
Chloride Mmol/l	80	65	76
Glucose Mmol/l	111	75	125

The current standard (hypo-osmolar) WHO ORS, with lower sodium and glucose content, was developed in order to reduce the intensity of diarrhoea in children. A meta-analysis of 8 trials, showed that reduced osmolarity ORS was associated with fewer unscheduled intravenous fluid infusions (the primary endpoint) compared with WHO standard ORS and stool output, reported in eleven trials, was less in the reduced osmolarity ORS group ^17^. Hypo-osmolar ORS has now been adopted into practice and has largely replaced the old ORS formulation and is recommended in current WHO paediatric management guidelines ^12^. The Standard (hyposmolar) WHO ORS recommended for non-SAM children is therefore closer in composition to ReSoMal with respect to sodium content but has a much lower osmolarity (due to the lower glucose content) than ReSoMal which means theoretically that it may have less potential to exacerbate stool volume and diarrhoea in children with SAM.

The ‘strong’ recommendations for rehydration of children with SAM are informed by a nutritional specialist group for the WHO and are based on expert opinion, since the review of the data indicated low quality of evidence ^6^. The most recent updates to WHO guidelines in 2013 did not revise any of their recommendations, with the exception of the addition of a single 15ml/kg bolus of hypotonic intravenous fluid for severely dehydrated children unable to tolerate oral rehydration (as per shock management) ^6^. No further IV rehydration beyond this was considered, with most rehydration strategies focused on oral rehydration. Owing to the poor outcomes recognised in African children with SAM complicated by diarrhoea, we therefore conducted a systematic review of the current available evidence underlying oral rehydration solutions for children with dehydration and severe acute malnutrition.

### Objectives

To conduct a critical appraisal of available evidence evaluating the use of ReSoMal and hypo-osmolar ORS in the treatment of dehydration in children with SAM.

## Methods

We did not publish a protocol prior to conducting this review. A search of online literature was performed. There were pre-determined criteria, as detailed below for eligibility of studies, data outcomes, and an assessment of risk of bias and study method quality in each of the identified studies.

### Selection criteria

***Population.*** Children aged 0 to 12 years with SAM requiring oral rehydration solution for management of dehydration secondary to gastroenteritis. We used the WHO definitions for malnutrition (Weight-for-height Z score (WHZ) <-3, mid-upper arm circumference (MUAC) <115mm or oedema consistent with kwashiorkor), gastroenteritis (dehydrating diarrhoea, >3 loose stools per day) and for dehydration. We excluded studies with chronic or persistent diarrhoea lasting ≥ 14 days.

***Intervention and comparison.*** All studies that compare two or more different ORS were included. Studies were excluded if they considered rehydration in children without severe malnutrition, only considered rehydration via the IV route or only included patients with congenital heart disease, trauma, or diabetic ketoacidosis.

***Outcome.*** Clinical trials that reported on any outcomes were included. The primary outcome for this review was frequency of hyponatraemia (sodium concentration <135mmol/L) during and after rehydration therapy. Secondary outcomes were all cause mortality, time to rehydration, stool output, frequency of fluid overload and frequency of oral rehydration failure.

***Study design.*** Only randomised-controlled trials (RCTs) were included.

### Search methods

***Online database search.*** A comprehensive literature search of the following databases was conducted on the 18 ^th^ July 2017 using the English search terms ‘malnutrition’ AND ‘children’ AND ‘rehydration’ AND ‘oral’:

- PubMed/ Medline
- Global Health Library (Virtual Health Library)
- Cochrane Database of Systematic Reviews
- Cochrane Central Register of Controlled Trials
- ClinicalTrials.gov
- The WHO International Clinical Trials Registry Portal (ICTRP) search portal

Each of the eligible studies was assessed and a manual review of the reference lists carried out. Additionally, a Google search was performed.

The authors screened the results of the literature search for studies that met the inclusion criteria as determined by the PICOS outline.

## Results

### Study selection

**Figure 1.**
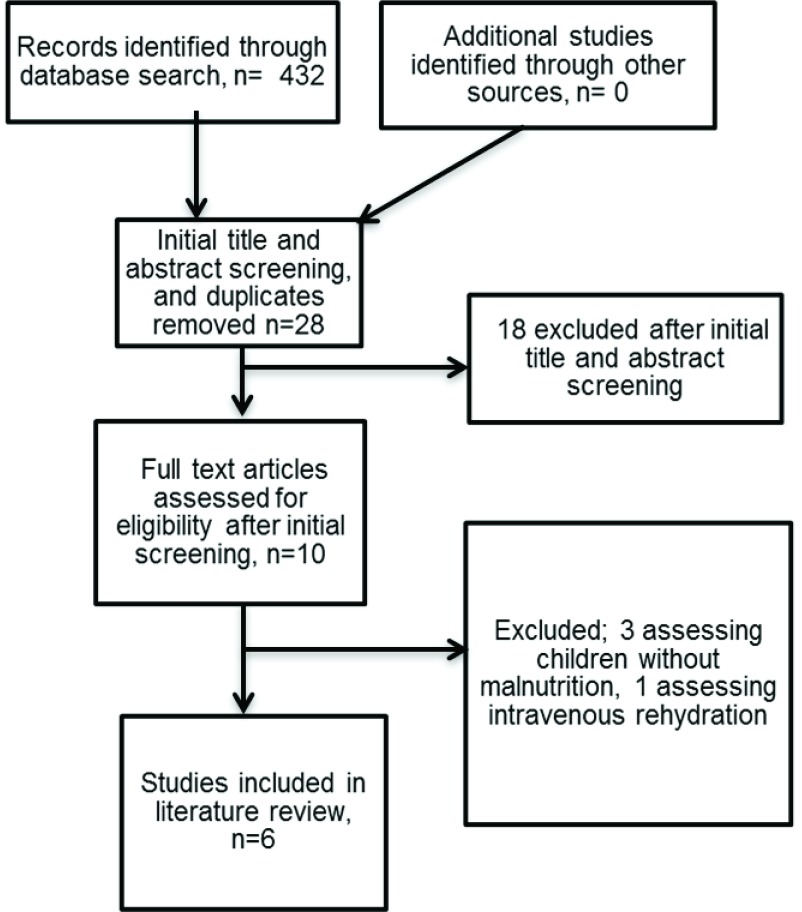
Flow diagram for selection of studies and reasons for study exclusion.

The search produced 432 studies ( Figure 1). After screening and evaluation, six studies were identified that investigated ORS in children with SAM complicated by dehydration, incorporating a total of 686 children. All six of these studies were conducted in Asia, four of which were conducted at the International Centre for Diarrhoeal Disease Research, Bangladesh (ICDDR, B) ^18– 21^ and two in India (New Delhi ^22^ and Calcutta ^23^). One study included children with and without SAM ^18^, but reported independently on outcomes for children with SAM. One study included children with cholera only ^20^ (see Box 1 for further details). There was moderate heterogeneity in the population eligibility criteria, sample size, and methods employed by each study, and in their results. Table 3 and Table 4 show the setting, methodology and features of the included studies and their results. Table 5 shows the formulations of ORS used in the studies.

**Box 1.** Management of cholera in children with SAM.

WHO guidelines	Children with SAM should be rehydrated slowly, either orally or by nasogastric tube, using WHO standard oral rehydration solution (ORS), 5ml/Kg every 30 minutes for the first 2 hours and then 5–10ml/Kg/hour up to a maximum of 10 hours. ReSoMal should not be given if the child has suspected cholera or has profuse watery diarrhoea. Intravenous fluid should not be used unless the child has shock and cannot be rehydrated orally or by nasogastric tube.
Evidence for oral rehydration	This review includes 259 (out of a possible total of 665) patients that had cholera (39%) in five of the six studies (Kumar *et al.*, 2015, did not report on numbers with cholera). One study included only children with cholera and two further studies presented sub-analyses on patients with cholera. Relevant findings: Alam *et al*. (2009) ^20^ included ONLY patients with cholera and reported that the only significant difference was a reduction in stool output with children receiving the rice-based ORS. Alam *et al*. (2000) ^18^ reported no significant difference in frequency of hyponatraemia. and significantly less hypo-osmolar ORS consumed than standard ORS. Alam *et al*. (2003) ^19^ showed no significant differences in hyponatraemia between patients with cholera treated with ReSoMal or standard ORS. Notably, the patient who developed hyponatraemic seizures did not have cholera.
Implications in practice	There appears to be no additional significant benefit to using hypo-osmolar ORS or ReSoMal in comparison with standard WHO ORS. Guidelines for children with SAM and suspected or confirmed cholera are identical to those with non-cholera diarrhoea other than the ORS used. Cholera is a secretory diarrhoea with high stool volume output and the current restrictive guidelines may therefore result in under treatment of children with dehydration.

**Table 3.** Characteristics of Included Studies.

	Year	Location	Study type	Population	Sample size	Inclusion	Exclusion	Comparison (number in group)	Outcomes
**Alam *et al.*^18^**	2000	ICDDR, B, Dhaka Hospital	RCT (Double blinded)	Children aged 3 months and above	170 Including 81 children with SAM	Children with acute (<4 days duration) diarrhoea with dehydration and one of the following: i) non- cholera diarrhoea, aged 3 months to 5 years; and ii) all children above 3 months with clinical suspicion of cholera	Children with evidence of systemic infection, encephalopathy, electrolyte imbalance, convulsion or invasive diarrhoea	Old WHO ORS (40) versus Hypo-osmolar WHO ORS (41)	Time to rehydration, stool frequency and duration of diarrhoea, time to discharge
**Alam *et al.*^19^**	2003	ICDDR, B, Dhaka Hospital	RCT (Double blind)	Children aged 6 to 36 months	130	Children with severe malnutrition (WFH <70% NCHS median or bilateral pedal oedema) and acute watery diarrhoea (<10days)	Children with bloody diarrhoea, severe dehydration requiring IV fluids or signs of severe infection	ReSoMal (75) versus old WHO- ORS (75)	Primary: Frequency of over hydration and number who corrected hypokalaemia at 24 ad 48 hours Secondary: number of children remaining hyponatraemic at 24/48 hrs
**Alam *et al.*^20^**	2009	ICDDR, B, Dhaka Hospital	RCT (Not blinded due to visible differences in the ORS)	Children aged 6 to 36 months	175	Children with severe malnutrition (WFH<70% NCHS median) and cholera (stool dark field microscopy)	Children with dysentery, and severe infections	Glucose-ORS (58) vs. Glucose-ORS plus 50g/L Amylase resistant starch (59) vs. Rice-ORS (58)	Primary: stool output Secondary: time to attain oedema free weight for length of 80%.
**Alam *et al.*^21^**	2015	ICDDR, B, Dhaka Hospital	RCT (double blind)	Children aged 6 to 36 months	126	Children with severe malnutrition (WFH/WFA <-3 Z score with or without pedal oedema) with acute diarrhoea (3 or more watery stools for 24 hours, lasting <7days)	Children with bloody diarrhoea, severe diseases (sepsis, meningitis, severe pneumonia)	Hypo-osmolar ORS with (63) and without (63) additional 15g/L PHGG (partially hydrolysed guar gum)	Duration of diarrhoea, proportion recovered within 72hours, daily stool output and recovery from severe malnutrition
**Dutta *et al.*^23^**	2001	Dr BC Roy Memorial Hospital, Calcutta	RCT (double blind)	Children aged 6 to 48 months	64	Male children <60% Harvard standard WFA without oedema, and history of acute water diarrhoea for <72hours and ‘some dehydration’	Children with recent history of diarrhoea (<1month), previous antibiotics or ORS during this episode, obvious parental infection, exclusively breastfed and if they had kwashiorkor	Hypo-osmolar ORS (32) versus Old WHO ORS (32)	
**Kumar *et al.*^22^**	2015	Kalawati Saran children’s Hospital, New Delhi	Open RCT	Children aged 6–59 months	110	Children with severe acute malnutrition as per WHO case definition.	Children with shock, oliguria (6hrs or more), convulsions and severely deranged electrolytes. Children with known renal, heart disease and recent IV fluid treatment.	Low-osmolarity ORS with added potassium versus modified ReSoMal	Primary: Number of children developing hyponatraemia. Secondary: frequency of hypokalaemia, treatment failure, stool frequency, ORS consumption and time to rehydration.

**Table 4.** Methodology and results of included studies.

	Risk of bias	Methodology	Frequency of hyponatraemia	Other outcomes (mortality, time to rehydration, time to discharge, stool output, frequency of fluid overload and frequency of treatment failure)
**Alam *et al.*,** **2000 ^18^**	Low- moderate	Serially allotted the study ORS packet (pre- made and ordered by pharmacy according to randomisation) – randomisation process not specified. Outcomes not pre-specified. If severely dehydrated they received 50ml/Kg RL in 1 hour before inclusion in the study. Then 75ml/kg ORS over 4 hours. Serial clinical assessments of dehydration.	No significant difference in serum sodium	Shorter duration of rehydration in hyposmolar ORS group although not significant (10.95 hours vs. 11.72 hours, p=0.32, 95% CI 0.55-0.97) Stool frequency during rehydration was less (4.27 vs. 5.86, p<0.05) Number of patients failing oral rehydration therapy not reported
**Alam *et al.*,** **2003 ^19^**	Low	Children enrolled and randomised (list provided by WHO Geneva and serially numbered). Blinding well detailed. Clinical history taken, blood, and stool samples taken, plus urine and CXR if indicated. WHO standard protocol followed. Children received 10m/Kg/h first 2 hours and then 5ml/Kg/hour over 10–12 hours until deficit corrected. On-going stool losses also accounted for.	3 children in the ReSoMal group developed severe hyponatraemia by 24 hours and one had hyponatraemic convulsions (serum sodium 108mmol/L) Serum sodium was lower at 24 and 48hrs in ReSoMal group (p<0.01 and p<0.001 respectively	Children equally and adequately well hydrated (ReSoMal vs. WHO-ORS, 76% vs. 81%, p-0.68) Number developing over hydration not significantly different (ReSoMal vs. WHO-ORS 5% vs. 12%, p=0.2) ReSoMal corrected basal hypokalaemia in greater proportion by 24 hours (36% vs. 5%, p=0.0006) and 48 hrs (29% vs. 10%, p=0.017 1 and 3 patients from the old WHO-ORS and ReSoMaL groups required IV rehydration after randomisation (no p-value given)
**Alam *et al.*,** **2009 ^20^**	Low	Severely dehydrated children were administered 100ml/Kg IV ‘cholera-saline’ for 4–6 hrs prior to randomisation. If eligible, children randomised to one of 3 ORS solutions (list prepared by independent statistician). Clinical assessment at admission, blood, urine and stool samples obtained. ORS 100ml/KG over 6 hours, plus additional ORS if high purging rate. Continued until cessation of diarrhoea. Standard protocol for SAM followed. Followed up for minimum 6 weeks.	Serum sodium at baseline not significantly different and subsequent hypo/hypernatraemia not reported	Time to rehydration not significantly different between groups All were rehydrated within 6 hours None developed over-hydration or heart failure Stool output and ORS intake in first 24 hours was significantly less in rice ORS group (p=0.004 and p=0.002 respectively) Urine output at 12 hours not significantly different Duration of diarrhoea and time to attain 80% median weight/length not different No significance difference in treatment failure (p=0.785)
**Alam *et al.*,** **2015 ^21^**	Low	Eligibility confirmed and then child randomised 1:1 to ORS (list provided by independent statistician and held by pharmacists). Children with severe dehydration were treated with IV fluids and then randomised as soon as they were out of hypovolaemic shock and signs of severe dehydration resolved (but <4hours). Clinical assessments, blood and urine samples. Otherwise followed WHO protocol.	Serum sodium at baseline not significantly different and subsequent hypo/hypernatraemia not reported	Mean duration of diarrhoea significantly shorter in PHGG group (p=0.01) Stool weight reduced in PHGG group, but not significantly different Mean time to attain WFL 80% was shorter in PHGG group (p=0.027) Time to rehydration not reported No significance difference in treatment failure (p=0.69)
**Dutta *et al.*,** **2001 ^23^**	Low- moderate	Eligibility checked, consented and clinical assessment done. Computer generated randomisation table used for allocation of ORS, held by independent individual who provided ORS packets. Blood and stool samples taken. Outcomes not pre-specified	Mean serum sodium on recovery was within normal range in both groups	Total of 29 (91%) in old WHO ORS group and 32 (100%) in hypo-osmolar ORS group recovered within 5 days. Stool output (52.3 v 96.6 g/kg/day) significantly less in hypo-osmolar group Duration of diarrhoea (41.5 v 66.4 hours) significantly less in hypo-osmolar group ORS intake (111.5 v 168.9 ml/kg/day) significantly less in hypo-osmolar group Fluid intake (214.6 v 278.3ml/Kg/day) significantly less in hypo-osmolar group Percentage weight gain in hypo-osmolar group was significantly less
**Kumar *et al.*,** **2015 ^22^**	Low	Enrolment, randomisation (block randomisation by computer generated sequence), then clinical assessment, and blood samples taken. Other treatment as per WHO-guidelines. Statistical analysis specified	Greater proportion of children developed hyponatraemia in ReSoMal group (15.4% vs. 1.9%, p=0.03)	Time for achieving rehydration was earlier in ReSoMal group (16.1 vs. 19.6h, p=0.036) Median stool frequency similar between groups Frequency of hypokalaemia similar (ORS vs. ReSoMal, 9.6 vs. 17%, p=0.25) Amount of ORS consumed was lower in ReSoMal (p=0.06) Both had equal number of successful rehydration No difference in treatment failure (p=1.0)

**Table 5.** Formulations of ORS used in Included studies.

	Modified WHO ORS ^&^	Hypo- osmolar ORS	Modified ReSoMal	Glucose- ORS	Glucose- ORS and ARS	Rice- ORS	Hypo- osmolar ORS	Old WHO ORS	Standard (hypo- osmolar) WHO ORS	ReSoMal
Osmolarity (mOsm/L)	302	245	300	305	305	215	224	311	245	300
Sodium mmol/l	75	75	45	75	75	75	60	90	75	45
Potassium Mmol/l	40	20	X	40	40	40	20	20	20	40
Chloride Mmol/l	87	X	X	87	87	87	50	80	65	76
Glucose Mmol/l	90	X	X	90	90	0	84	111	75	125
Rice Powder g/L	0	0	0	0	0	50	0	0	0	0
ARS ^*^, g/L	0	0	0	0	50	0	0	0	0	0
PHGG ^#^ g/L	15	0	0	0	0	0	0	0	0	0
Used in study	Alam *et al.*, 2015 (+/- PHGG)	Kumar *et al.* 2015		Alam *et al.* 2009			Dutta *et al.* 2001		Alam *et al.* 2000	Alam *et al.* 2003 vs WHO ORS

^*^ARS – Amylase-Resistant starch ^#^PHGG – partially hydrolysed guar gum ^&^ORS - Oral rehydration solution - not presented in paper

### Risk of bias

The quality of each of the included studies was assessed for risk of bias using the Cochrane collaboration’s tool in order to evaluate validity. Four studies had a low risk of bias ^19– 22^ and two had low-moderate risk of bias ^18, 23^ due to lack of pre-determined outcomes in the methods.

### Outcomes

***Primary outcome***

*Hyponatraemia*

This outcome was available from four studies. Two of these compared old WHO ORS formulations with hypo-osmolar formulations (Alam *et al.* 2000 and Dutta *et al.* 2001). Alam *et al.* (2000) only reported baseline chemistry ^18^; however Dutta *et al.* (2001) found no significant differences in sodium at baseline and recovery, with sodium levels remaining within normal limits at recovery for both formulations of ORS ^23^. Two studies (Alam *et al.* 2003 ^19^ and Kumar *et al.* 2015 ^22^) compared ReSoMal with old WHO ORS: the first (Alam *et al.* 2003) compared ReSoMal with Old WHO ORS and found that 1/64 (2%) in the ORS group developed severe hyponatraemia (Na≤120mmol/L) compared to 3/62 (5%) children receiving ReSoMal, with one of these three experiencing hyponatraemic seizures (serum sodium 108mmol/L). Serum sodium was similar at baseline in both arms (p=0.51), but was lower at 24 and 48 hours in ReSoMal group (p<0.01 and p<0.001 respectively). The second (Kumar *et al.* 2015) compared ReSoMal with hypo-osmolar ORS and found that a greater proportion of children in the ReSoMal group developed hyponatraemia (15.4% vs. 1.9%, p=0.03).

***Secondary outcomes***

*Mortality*

This outcome was reported in two studies. Alam *et al.* (2003) ^19^ and Alam *et al.* (2009) ^20^ reported no deaths during the trial periods.

*Time to rehydration or recovery*

Five of the studies reported on time to rehydration. One study (Alam *et al.* 2009) ^20^ found that there was no significant difference in time to rehydration (time to attain 80% of weight for length or height) between groups. Two studies evaluating old WHO ORS versus hypo-osmolar ORS (Alam *et al.* 2000 ^18^ and Dutta *et al.* 2001 ^23^) found that there was a faster recovery (passage of 2 consecutive formed stools or no diarrhoea for 12 hours) in the group receiving hypo-osmolar ORS compared to old WHO ORS (36 vs. 53 hours, p=0.001). Alam *et al.* (2000) reported average time to rehydration (though how this was assessed was not defined) of 10.95 hours in hypo-osmolar group versus 11.7 hours in old WHO-ORS group (p=0.32). Dutta *et al.* (2001) reported faster time to recovery in hypo-osmolar group. Of the two studies comparing ReSoMal with WHO ORS (old or hypo-osmolar), one study reported shorter time to rehydration in ReSoMal group (Kumar *et al.* 2015) ^22^ (16.1 hours compared with 19.6 hours in group receiving hypo-osmolar ORS, p=0.036), whilst the Alam *et al.* (2003) trial reported that the groups were equally well rehydrated by both regimes ^19^.

*Stool output*

All of the studies reported on stool output, either by measuring weight of stools or nappies (Alam *et al.* 2003, 2009, 2015 and Dutta 2015) ^19– 21, 23^ or recording frequency (Alam *et al.* 2000 and Kumar *et al.* 2015) ^18, 22^. Of the two studies comparing standard with hypo-osmolar ORS, both found that the stool output was significantly less in the group receiving hypo-osmolar ORS (Alam *et al.* 2000 found that daily stool frequency was 4.27 compared with 5.86 episodes, p<0.05, and Dutta *et al.* (2001) found that stool output was 52.3 versus 96.6 g/Kg/day, p=0.0001). Alam *et al.* (2009) and (2015) found that stool weight (collected in a bucket or in pre-weighed nappies and weighed) was significantly less when children received rice-based ORS (compared with glucose based ORS) or partially hydrolysed guar gum (PHGG) added to ORS (compared with hypo-osmolar ORS). Alam *et al.* (2000) and Kumar *et al.* (2015) reported that stool frequency was similar between the ReSoMal and hypo-osmolar ORS groups.

*Frequency of fluid overload*

Three studies reported on frequency of fluid overload. Alam *et al.* (2003) ^19^ reported no significant difference in over-hydration with each of the three formulations of ORS (defined as >5% weight gain after correction of dehydration at any time during the study period with any of the following signs: periorbital oedema/puffy face, increased heart rate (>160/min) or increased respiration (>60/min)). Both Alam *et al.* (2009) ^20^ and Kumar *et al.* (2015) ^22^ did not report signs of fluid overload.

*Oral rehydration failure*

This outcome was available with varying definitions from the 5 studies. Alam *et al.* (2003) ^19^ and (2015) ^21^ Kumar *et al.* (2015) ^22^ reported the number of patients requiring IV fluids after randomisation. There were no significant differences in this outcome between groups receiving ORS (hypo-osmolar or old WHO) and ReSoMal, nor did addition of PHGG to ORS reduce the treatment failure rate). Alam *et al.* (2009) ^20^ defined failure as on-going diarrhoea 7 days after randomisation and found similar numbers (one and two patients from the glucose-ORS and glucose-ORS plus ARS groups, respectively) in each group. Dutta *et al.* (2001) ^23^ reported the number of patients who failed to recover after 5 days and again found no difference between groups receiving hypo-osmolar and old WHO ORS.

## Discussion

These studies evaluated a number of different combinations of ORS formulations, including old WHO ORS (sodium level 90mmol/L), standard/current WHO hypo-osmolar ORS (sodium 75mmol/L) and ReSoMal (sodium 45mmol/L), with minor variations within the composition across studies (PHGG and Amylase-Resistant Starch). All studies used conventional definitions for diagnosis of severe acute malnutrition, i.e. using WHO or NCHS criteria, and varying criteria for assessment of severity of dehydration. All of the study interventions were blinded to patients and clinicians except in the Kumar *et al.* (2015) ^22^ and Alam *et al.* (2009) trials. This is important to mention as strength of the design of the trial, since a number of the outcomes were subjective, in particular, the secondary outcomes: time to rehydration or recovery, frequency of fluid overload and treatment failure. However, Kumar *et al.* (2015) used frequency of hyponatraemia as the primary outcome - an objective, quantitative outcome. Notably, none of these studies assessed ORS therapy in the community, and all the reported studies have been conducted in Asia, and in none of the trials were there any case fatalities. Of note, none of the trials were conducted in African children, who have a much higher mortality rate when SAM is complicated by diarrhoea ^1^.

There was no improvement in frequency of hyponatraemia with rehydration when comparing current standard WHO hypo-osmolar with old WHO ORS. There were no differences in between-group rehydration failure rates within any of the studies; no rehydration solution demonstrated superiority for this outcome. However, use of standard hypo-osmolar ORS appeared to have advantages in terms of reduction of stool output, duration of diarrhoea and time to rehydration, when compared with old WHO ORS.

Use of ReSoMal, when compared with old WHO ORS and standard hypo-osmolar WHO ORS resulted in greater proportions of children developing or worsening of hyponatraemia after rehydration treatment ^22^. In one study, this was associated with severe hyponatraemia in three children and development of hyponatraemic seizures in one of these children ^19^. ReSoMal did however correct hypokalaemia in a greater proportion of children and in a shorter timeframe (serum potassium at 24 hours in ReSoMal group 4.0 vs. 3.2 in WHO-ORS group, p=0.001) ^19^. ReSoMal also shortened time to rehydration in one study comparing ReSoMal with hypo-osmolar ORS ^22^.

As noted by one of the reviewers we included in the systematic review two trials that compare oral rehydration solutions with identical electrolyte contents (thus do not directly address the question of electrolyte/osmolarity) but are worthy of specific mention. Included in the first trial by Alam *et al.*, 2009 as a comparator to standard ORS were two additional arms with either amylase-resistant starch or rice added to the glucose ORS ^20^. The second trial by *Alam et al.*, 2015 compared hyposmolar WHO ORS to hyposmolar WHO ORS with added partially hydrolysed guar gum) ^21^. The hypothesis being tested in these trials was whether these fermentable carbohydrates, that form short chain fatty acids in the colon, could improve gut barrier function by providing energy to the colon and improving overall metabolism and reduce the duration of diarrhoea. These have the potential to reduce overall stool volume and recovery time and could be considered as candidates for future trials in African children with mortality as a key endpoint.

Unlike the studies reported in Asia, diarrhoea comorbidity in African children hospitalised with SAM has a poor prognosis, with a case fatality rate of 18–20% ^1^. A large prospective study investigating risk factors for mortality in 1206 Kenyan children with SAM and diarrhoea at admission to hospital (≥3 watery stools/day) showed that both hyponatraemia and hypokalaemia were associated with a greater risk of mortality: hyponatraemia odds ratio 4.6 (95% CI 2.0,10.6, p<0.001) and hypokalaemia odds ratio 2.5 (95% CI 1.3, 4.6, p<0.004) ^1^. Hyponatraemia has nearly twice the impact on risk of mortality when compared with hypokalaemia; therefore, it would be prudent to place the importance of sodium status ahead of potassium. By this deduction, there is no clear advantage of ReSoMal over standard or hypo-osmolar rehydration solutions in terms of sodium status. Conversely, there are risks of serious harm through development of symptomatic hyponatraemia.

These findings highlight the lack of compelling evidence to support the current rehydration guidelines for management of children with SAM complicated by diarrhoea. Hypo-osmolar solutions have no apparent benefit on sodium status, i.e. there is no significant difference in numbers with hyponatraemia after rehydration, but do have significant improvements on reduction of stool volume and frequency, and on duration of diarrhoea. It is unclear, however, whether this translates to a survival advantage. Just two of the studies reported on mortality, and no deaths were observed in either. The mortality rate in children with SAM and diarrhoea in African children is substantially higher than reported from the studies included in this review. It would therefore be useful to conduct similar trials in children in the African continent.

### Reappraisal of current guidelines

The WHO reviewed the guidelines for management of SAM in 2013 and identified five papers (four of which have been included in this review). The review discusses evidence presented by the papers and, despite universally ‘low quality of evidence’, continues to make ‘strong recommendations’: choosing only to amend the SAM management guidelines to allow the use of hypo-osmolar ORS and advise that ‘either ReSoMal OR half strength standard WHO low-osmolarity oral rehydration solution with added potassium and glucose should be given’ to children with some or severe dehydration. The review also emphasises that ReSoMal ‘should not be given if children are suspected of having cholera or profuse watery diarrhoea’.

### Application of guidelines

In practice, according to the WHO guidelines for management of children with SAM and diarrhoea, after the first two hours ReSoMal should be alternated hourly with F75, a specialised feeding formula for children with SAM (see Table 1). These are largely based on expert opinion and not on experimental evidence. The rehydration guidelines do not offer a pragmatic or evidence based approach to management of children with SAM complicated by dehydration and they are open to wide interpretation and misuse. Furthermore, for undernourished children with severe dehydration (equivalent of 10% or more loss of body weight), up to 20% of children hospitalised with gastroenteritis fulfil SAM anthropometric criteria for SAM (MUAC <11.5cm or WHZ <-3SD), but following rehydration they are reclassified as undernourished. Thus, the current recommendations have much wider implications with many ineligible children receiving potentially harmful low sodium rehydration solutions ^24^.

## Conclusions

The available evidence for management of children with SAM and dehydration is limited and does not lend support to the WHO guidelines. There are arguments to support the use of hypo-osmolar ORS in children with SAM, but the currently recommended ReSoMal exposes children with SAM to risk of severe hyponatraemia. Further research should evaluate use of standard hypo-osmolar ORS in children with SAM, and assess optimal rates of rehydration in order to construct evidence based pragmatic guidelines that are designed for the context in which they will be used. In particular, it would be useful to conduct research in sub-Saharan Africa, given that none of the available evidence relates to this population.

## Supplementary material

Supplementary File 1: PRISMA checklist.
