# Supplementary material for: Oral rehydration of malnourished children with diarrhoea and dehydration: A systematic review
Source: Wellcome Open Res. 2017 Oct 27;2:66. Originally published 2017 Aug 18. [Version 3] doi: 10.12688/wellcomeopenres.12357.3 (PMC5657219; doi:10.12688/wellcomeopenres.12357.3)
Supplement: Peer review history

# Peer review: Referee response for version 2

**DOI:** 10.21956/wellcomeopenres.13842.r26515

**Published:** 2017-10-20

**Authors:** André Briend

**Affiliations:** 1 Department of Nutrition, Exercise and Sports, University of Copenhagen, Frederiksberg, Denmark

This version of the manuscript is improved compared to the previous one and the author did take into acount the points I made.

 Two few minor points need correction:

 a) In the discussion, the authors say:

 All of the study interventions were blinded to patients and clinicians except in the Kumar et al. (2015) trial.

 The study by Alam et al (2009) was not blinded either. See the following sentence in the method section of this paper.

*“The treatment could not be blinded to the people involved in the study because of visible differences in the ORS solutions”.*

 Table 3 describes the same study as double blinded. .This should be corrected as well.

 b) Further in the discussion the authors say:

 “However, Kumar et al. (2015) used frequency of hyponatraemia as the primary outcome - an objective, qualitative outcome”.

 Do they mean – an objective quantitative outcome?”

I have read this submission. I believe that I have an appropriate level of expertise to confirm that it is of an acceptable scientific standard.

# Response

**Published:** 2017-10-23

**Authors:** Kathryn Maitland

**Affiliations:** KEMRI-Wellcome Trust Research Programme,, Kenya

Thank you Professor Briend. We have made the suggested changes and have submitted the revised version (3).

# Peer review: Referee response for version 1

**DOI:** 10.21956/wellcomeopenres.13380.r25181

**Published:** 2017-09-04

**Authors:** Maren Johanne Heilskov Rytter

**Affiliations:** 1 Department of Nutrition, Exercise and Sports, University of Copenhagen, Frederiksberg, Denmark

**Comments to the authors:**

 This is an welcome, interesting and well-written review illustrating the sparse evidence underlying the current WHO recommendations for rehydration in children with SAM (as other treatments for SAM!), and points to important future studies to be done.

**General comments:**

 The fact that no children died in the reviewed studies illustrates that the study population of Asian children is very different from the population of interest mentioned in the introduction (children with SAM and diarrhoea in Africa, with high mortality).  This issue is discussed, but in addition to the differences already mentioned, it may be relevant to mention that Asian children more frequently have marasmus and less frequently kwashiorkor. Cholera is also likely to be more frequent in Asia (requiring higher Na in rehydration fluids), and HIV is less prevalent. This is likely to make SAM+ diarrhoea a totally different disease complex in African than in Asian children. This does not make the review less relevant, but underlines the conclusion that we know very little about what we do when treating very sick children.

 It may be informative to explain the history of development of ReSoMal, “Standard-WHO-ORS” and “low-osmolarity ORS” (now endorsed by WHO, and therefore by most health staff considered Standard ORS), which (at least to me) has been a little confusing:

 Standard ORS was mainly developed to treat cholera in the first place, and therefore had a relatively high Na content.

 ReSoMal was developed at a time when this high-Na ORS was used, due to  (theoretical) concerns about malnutrition being prone to Na retention, greater K deficit and higher risk of hypoglycaemia in children with SAM (particularly in oedematous children), but as emphasised it was never formally tested whether it was indeed better.

 Low-osmolar ORS was developed to reduce the intensity of diarrhoea in children, and has been shown to be successful in that respect  (see https://www.ncbi.nlm.nih.gov/pubmed/11869639 ^1^) (although probably less appropriate for children with cholera, in whom Na loss is higher), and in most parts of the world low-osmolar ORS is now standard treatment for children with diarrhoea (and no malnutrition).

 This means that the ORS used for normal children is now closer in composition to ReSoMal regarding Na content than when this was developed. Notably, it also means that the osmolarity of ReSoMal is now *higher* than the ORS used for well nourished children,potentially increasing stool output in already very vulnerable children.

 Another very practical problem with ReSoMal is what to do when ReSoMal is not available, which is the case for many primary care health facilities. Well-trained health staff will have learned that ORS should NEVER be given to malnourished children. Even though the WHO protocol has a recipe for ReSoMal made from ORS (including addition of potassium) my guess is that this “barrier of complication” makes necessary rehydration therapy unavailable for many children with SAM and dehydration seen in primary care.

 As highlighted by the authors the evidence base underlying WHOs guidelines for SAM is generally poor, with few RCTs and a lot of expert opinion and pathophysiologic speculations. Some of the least poor evidence is  (in my opinion) is a from semi-observational studies where outcomes were compared in hospital facilities before and after implementation of a protocolised treatment package with some elements similar to the WHO guidelines. One of these, a study from Bangladesh compared outcomes before and after implementation of a treatment protocol for children with diarrhoea and SAM including early milk feeding, empiric antibiotics, emphasis on oral rehydration and slower initial rehydration. They found that children given protocolised treatment had almost half the risk of dying compared to children treated before the protocol was implemented. I know that your inclusion criteria was limited to RCTs, and therefore this study is not eligible, but it may be worthwhile to include in the discussion. Worth noting is that the rehydration solution used in both periods contained 90 mmol/l of sodium, higher than currently used low-osmolarity ORS, indication that good improvements in outcome can be obtained by relevant interventions while still giving higher sodium ORS than suggested by WHO guidelines. See https://www.ncbi.nlm.nih.gov/pubmed/10371570 ^2^.

 A quick search for experimental /laboratory/animal study data supporting the “well-established fact” that children with malnutrition have tendency to Na retention. Except for children with kwashiorkor (in whom this tendency is very obvious) I haven’t found any, which was a bit of a surprise (although I must admit that my search may not have been extensive enough). I wonder if it would be worthwhile to ask the people who wrote the WHO manual if they knew of laboratory or experimental data supporting the idea of Na and fluid retention (in non-oedematous children)?

 I am a bit puzzled about the choice of primary outcome: The most important outcome should be mortality, or something else of direct clinical relevance (like: clinical deterioration, convulsions, failure of oral therapy ect). It is of course also relevant to report other outcomes (like laboratory tests), bearing in mind that these are always surrogate markers for relevant health outcomes. Other similar outcomes to report could be hypo-kalemia, and hypo-glycemia (if these were reported by any studies). Even though they (as argued) may not have equal clinical significance, they should be reported in the result section and not in the discussion (as hypo-kalemia is now).

 The studies associating hyponatremia with mortality are from Africa; and the included studies finding development of hyponatremia with rehydration are from Asia, where hyponatremia does not seem be associated with mortality (see for example: Chisti *et al:* Predictors of death in under-five children with diarrhoea admitted to a critical care ward in an urban hospital in Bangladesh, Acta Paediatrica 2011 ^3^). So the suggested evidence for a causal links between low-sodium rehydration and mortality is indirect and a bit weak weak… The causes for developing hyponatriemia in African children may be different from the causes of hyponatremia in Asian children. Also, I am not sure that hyponatremia was neccesarily a cause of death in the African children, although associated with mortality, but a sign of severe disease. It is possible that correcting their hyponatriemia would not make their prognosis better, but simply be “biochemical make-up”. Similarly, iatrogenic hyponatremia (although probably not a good thing) may not be caused by the same mechanisms as admission hyponatremia. Your concerns about hyponatremia is relevant, but maybe the weakness of surrogate markers should be discussed. The conclusion being be that a randomised trial among those who need better treatment the most is urgently needed. And with the high current mortality it is neccesary and realistic to use mortality as an outcome.

**Specific points:**

 Page 3, first paragraph:  It is not clear where the exact numbers come from:  49% with diarrhoea, 16% developing diarrhoea when admitted, mortality of 9% and 19%. It looks like they are derived from a specific study, but which one? Or are they averages from the four studies referenced? In any case I would not be so specific, but rather state that “..diarrhoea complicates around half of admissions for SAM…”

 It would be helpful if the sodium contents of the different solutions could be incorporated into table 3 (although they are given in table 5), to appreciate the comparison.

 Fluid therapy seems particularly tricky in children with oedema, and clinicians have varying approaches to this. In spite of this, the WHO protocol does not differentiate fluid therapy to oedematous and non-oedematous children, and is not very clear about diagnosing dehydration in these (overhydrated) children. It seems plausible that oedematous and non-oedematous children could require different strategies, but of course there is no evidence for this. In order to make this more clear future studies should report results separately for oedematous and non-oedematous children, and test any effect for interaction with oedema. Although I am aware that your included studies did not do this, it would be helpful if table 3 could specify how many children had oedematous malnutrition in each study?

I have read this submission. I believe that I have an appropriate level of expertise to confirm that it is of an acceptable scientific standard.

## References

[1] HahnSKimSGarnerP: Reduced osmolarity oral rehydration solution for treating dehydration caused by acute diarrhoea in children. *Cochrane Database Syst Rev*.2002; 10.1002/14651858.CD002847 CD002847 10.1002/14651858.CD002847 11869639

[2] AhmedTAliMUllahMMChoudhuryIAHaqueMESalamMARabbaniGHSuskindRMFuchsGJ: Mortality in severely malnourished children with diarrhoea and use of a standardised management protocol. *Lancet*.1999;353(9168) : 10.1016/S0140-6736(98)07499-6 1919-22 10.1016/S0140-6736(98)07499-6 10371570

[3] ChistiMJPietroniMASmithJHBardhanPKSalamMA: Predictors of death in under-five children with diarrhoea admitted to a critical care ward in an urban hospital in Bangladesh. *Acta Paediatr*.2011;100(12) : 10.1111/j.1651-2227.2011.02368.x e275-9 10.1111/j.1651-2227.2011.02368.x 21627690

# Response

**Published:** 2017-09-26

**Authors:** Kathryn Maitland

**Affiliations:** KEMRI-Wellcome Trust Research Programme,, Kenya

Please see our joint response

# Peer review: Referee response for version 1

**DOI:** 10.21956/wellcomeopenres.13380.r25180

**Published:** 2017-08-21

**Authors:** André Briend

**Affiliations:** 1 Department of Nutrition, Exercise and Sports, University of Copenhagen, Frederiksberg, Denmark

This is an important critical review of the evidence supporting the current WHO recommendation of using a low sodium high potassium solution (Resomal, 45 mmoles Na, 40 mmoles K) in children with diarrhoea and severe malnutrition. The paper is correct in highlighting that the strength of this evidence is very low, as acknowledged in the quoted 2013 WHO document. The conclusion of the paper, namely that a randomized study comparing the outcome of children treated with current low osmolarity WHO ORS and Resomal seems warranted. It could be argued however that with two randomised high quality studies suggesting that Resomal increases the risk of hyponatremia, and none showing a clear benefit beyond better correction of hypokalaemia, a change of recommendation could already be considered. It could be recommended to test the current low osmolarity WHO ORS with added potassium (as in the Alam *et al* 2009 study ^1^) vs. resomal.

 The paper has a clear focus on electrolyte disorders of children with diarrhoea and especially on the risk of hyponatraemia in relation to the sodium content of the rehydration solution. This is fine but in this case, one wonders whether two papers now included in the evidence base should not be excluded, namely the Alam 2009 ^1^ and Alam 2015 papers ^2^. These two papers compare oral rehydration solutions with identical electrolyte content, and are not really relevant to the hypothesis discussed here. These two papers describe an attempt to decrease stool output and to improve nutritional recovery by the addition of ingredients (resistant starch or PHGG) acting as soluble fibres. The idea is that these ingredients will not be digested in the upper gut and will be fermented in the colon to form short chain fatty acids which provide some energy to the colon and overall metabolism and may reduce the duration of diarrhoea and help to improve nutritional recovery. Their main outcome is duration of diarrhoea and their secondary outcome is time to nutritional recovery which is quite different from recovery from dehydration or correction of electrolyte disorders. I would be in favour of removing these two studies from the evidence base. This would leave a clearer message, namely that ORS with 75 mmoles/L of sodium does not increase risk of hyponatraemia compared with ORS with 90 mmoles/L whereas hyponatraemia seems to be a problem when sodium is as low as 45 mmoles/L as in Resomal.

 If the authors want to keep these two Alam *et al*. studies, they should broaden the discussion beyond electrolyte composition and discuss the evidence suggesting that some soluble fibres should be added as ingredient to the oral rehydration solutions.

 The statement that there is no data on the risk of sodium overload in children suffering from severe malnutrition needs qualification. The current concern is based on an old paper from Uganda reporting an excess of heart failure in children receiving a high energy milk to which sodium was added, apparently to improve acceptability (See: Wharton *et al.,* 1967 ^3^). It can be argued that the quantity of sodium given to these children was presumably higher than those receiving low osmolarity ORS, also that these children received this high sodium milk for much longer that ORS is given, and also they had a high sodium intake in absence of diarrhoea, which means these findings cannot be extrapolated to children with diarrhoea, but this paper should be quoted. Also, there are observations suggesting that even Resomal given liberally to children in absence of diarrhoea can lead to an excess mortality due to heart failure which could be due to excessive sodium intake (See: Grellety Y, 2000 ^4^). Arguably, this is again low quality evidence in favour of using low sodium rehydration solution in case of diarrhoea, but this should be mentioned as well.

   

 Minor comments

 The term “standard ORS” to refer to the old WHO solution (with 90 mmoles Na/L) is confusing, as this solution is no more recommended and the “low osmolarity ORS (Na 75 mmoles/L) is now the WHO standard. Choose another term avoiding the confusion, maybe just “old WHO ORS with 90 mmoles/L Na”.

 Abstract:

 Resomal contains 40 mmoles /L of potassium, not 20.

 Discussion:

 The authors say:

 “ReSoMal also shortened time to rehydration in one study comparing ReSoMal with hypo-osmolar  ORS”.

 Reference?

 “Unlike the studies reported in Asia, diarrhoea comorbidity in children hospitalised with SAM has a poor prognosis, with a case fatality rate of 18–20%.”

 Clarify. Do you mean in Africa ?

I have read this submission. I believe that I have an appropriate level of expertise to confirm that it is of an acceptable scientific standard, however I have significant reservations, as outlined above.

## References

[1] AlamNHIslamSSattarSMoniraSDesjeuxJF: Safety of rapid intravenous rehydration and comparative efficacy of 3 oral rehydration solutions in the treatment of severely malnourished children with dehydrating cholera. *J Pediatr Gastroenterol Nutr*.2009;48(3) :318-27 1927478810.1097/mpg.0b013e318180af27

[2] AlamNHAshrafHKamruzzamanMAhmedTIslamSOlesenMKGyrNMeierR: Efficacy of partially hydrolyzed guar gum (PHGG) supplemented modified oral rehydration solution in the treatment of severely malnourished children with watery diarrhoea: a randomised double-blind controlled trial. *J Health Popul Nutr*.2015;34: 10.1186/s41043-015-0003-3 3 10.1186/s41043-015-0003-3 26825757PMC5026008

[3] WhartonBAHowellsGRMcCanceRA: Cardiac failure in kwashiorkor. *Lancet*.1967;2(7512) :384-7 416633110.1016/s0140-6736(67)92006-5

[4] GrelletyY: Management of Severe Malnutrition in Africa. *(Doctoral thesis, University of Aberdeen)*.2000; Reference source
